# Data Delivery in a Disaster or Quarantined Area Divided into Triangles Using DTN-Based Algorithms for Unmanned Aerial Vehicles

**DOI:** 10.3390/s21113572

**Published:** 2021-05-21

**Authors:** Razvan Udroiu, Adrian Marius Deaconu, Corina-Ştefania Nanau

**Affiliations:** 1 Department of Manufacturing Engineering, Transilvania University of Brasov, 29 Eroilor Boulevard, 500036 Brasov, Romania; udroiu.r@unitbv.ro; 2 Department of Mathematics and Computer Science, Transilvania University of Brasov, 29 Eroilor Boulevard, 500036 Brasov, Romania; corina.nanau@unitbv.ro

**Keywords:** unmanned aerial vehicle (UAV) network, drone, delay tolerant networks (DTN), optimization algorithms, mobility schedule, quarantined areas, experimental flight

## Abstract

The communication in quarantined areas, e.g., due to the new COVID-19 pandemic, between isolated areas and in areas with technical damage has resulted in a great deal of interest concerning the safety of the population. A new method for ensuring communication between different areas, using unmanned aerial vehicle (UAV) networks with a well-established mobility schedule is proposed. UAVs fly based on a mission plan using regular polygons covering an area from a map. The area is considered to be equidistantly covered with points, grouped in triangles which are further grouped into hexagons. In this paper, UAVs, including battery charging or battery swapping stations and light weight Wi-Fi boards, are used for the data transfer among drones and stations using delivery protocols. UAV network analysis and evaluation (lengths of the arcs in seconds) based on experimental preliminary flight tests are proposed. Multiple simulations are performed based on six DTN algorithms, single-copy, and multiple-copies algorithms, and the efficiency of data transmission (delivery rate and latency) is analyzed. A very good delivery rate of 0.973 is obtained using the newly introduced TD-UAV Dijkstra algorithm.

## 1. Introduction

Nowadays, delay- and disruption-tolerant (DTN) networks are useful for providing communication in environments where frequent transmission discontinuities are present [1,2,3,4]. The need for DTN networks appeared initially in the field of space communication networks [1,2,3], but later, new applications of them appeared, such as underwater communications systems with high attenuation of radio waves, rural networks, environmental monitoring networks, vehicle networks [4,5], battlefield networks [6], intelligent transport systems, smart cities [7], etc. In DTN networks, devices update their communication routes based on the topological changes of the network. In this way, the mobility of the devices is one of the important aspects of a kind of network, detailed in [8,9,10]. The transmission mechanism for messages in this type of network is based on the store-carry-forward paradigm [11,12].

Routing [13,14,15] is the decision taken in the process of finding the best path to follow the messages through the network to reach the destination. The problem of routing in DTN networks is thus reduced to an optimization problem, constrained by the availability or unavailability of the edges in time and the storage capacity of each node in time. Several criteria classify the routing strategies used by the DTN networks, such as type of connection between nodes, the moment to establish the path for messages, the amount of information that the nodes have about the network, and the number of copies of a message that a node sends further. The type of connection between nodes is opportunistic connection based on a schedule, considering that in space everything is in motion and the nodes that can be connected move on a well-defined route, which implies the possibility of establishing its position at a certain time, as well as the possibility of establishing a connection based on this information. This connection is used for space networks and public transport networks. If we consider the moment of path establishing, the source node can calculate and set the path and then encode it inside the message, or each intermediate node calculates only the next message hop based on the information it has from the network [15]. A series of algorithms that contain different amounts of network information are proposed for investigation [15]. Among them, a variant of the Dijkstra algorithm is considered. The main change from the classic Dijkstra algorithm is that in order to determine the cost per arc, the time when the message reaches the node and the transfer time are taken into account. These values are used to calculate the cost of sending the message to the following link. It has been shown that the performance of these algorithms is gradually increasing, depending on the amount of information about the network they use. Considering the number of copies of a message, there are single copy algorithms (forward-based) and multi-copy algorithms (flood-based) [16,17]. Direct Delivery is a simple forward-based protocol, which involves delivering the message only to the destination node. This type of algorithm is suitable for small and high mobility networks, where the probability of meeting between nodes is high. Examples of classical flood-based protocols are Epidemic [18], Spray and Wait [19], PRoPHET [20], and MaxProp [21]. In [16] there are some comparisons between the first four of them. In the literature there are several variations of these algorithms. In buffer-limited delay-tolerant networks, maximum flow with a static approach can be considered [22]. The classic dynamic network can be adapted to the time-expanded network [23].

Spaho [24] investigated the performance of five routing protocols using an Opportunistic Network Environment (ONE) environment and simulated a data collection application using a DTN composed of pedestrians and cyclists equipped with smart devices. One of her conclusions was that MaxProp performs better than other protocols in terms of delivery probability, average latency, and buffer time. Li et al. identified the basic functionality requirements for DTN support comparing the Epidemic Routing and Named Data Networking [25]. They concluded that in the network storage, DTN communications critically depend on the store-carry-forward mechanism. Tikhonov et al. analyzed the effectiveness of the DTN system in a communication network on a railway line [8], showing a 20% reduction in message delivery time depending on the schedule of trains. Mao et al. proposed a routing protocol called Scheduling-Probabilistic Routing Protocol using History of Encounters and Transitivity (PROPHET) to improve the delivery rate and optimize delivery delay with low overhead in DTN for IoT applications [26]. Their performed simulations in the ONE simulator showed that the Scheduling-PROPHET routing protocol achieved a high delivery rate and reduced delivery delay while minimizing resource consumption in transmission.

The UAVs with their flexibility are popular and have gained importance within many applications across many domains such as atmospheric sciences [27,28,29], communications [26,30,31,32], transportation (delivery) [33,34], agriculture [35], cartography [36], rescue missions [37], etc. The UAV applications are summarized in Table 1, which shows whether the UAVs are grouped into networks. In the literature, there are very few papers considering networks consisting of regular polygons. In addition, most of the studies do not specify the drone type.

Rango et al. proposed a bio-inspired coordination protocol for UAV flying ad-hoc network (FANET) management in the agriculture domain [35]. The proposed system has been simulated in an ad hoc simulator, and a preliminary analysis about the feasibility of the UAV design for the specific purpose has been performed. Hu et al. investigated the UAV-based air-to-ground wireless networks and evaluated the performance of the branch and bound search-based mode selection (BBS-MS) [30].

Vehicular ad hoc networks (VANETs), which are a type of mobile ad hoc network (MANET), play the most important role in an intelligent transport system. Within the VANET, the mobile vehicles can establish three main categories of communications: vehicle to vehicle, vehicle to infrastructure, and infrastructure to infrastructure. A relative recent application of VANET is for a UAV network that can communicate for message relaying with each other via wireless links [31].

The UAV flight path planning problems can be categorized as off-line planning, on-line planning, and cooperative planning [38,39]. The limited battery energy of UAVs [40] allows them to fly for a relatively short distance. Using UAV networks, a large area can be covered. Rosa et al. proposed a space mapping method using a topological map as a collection of hexagons; each hexagon can hold a maximum of one UAV [37]. The simulations in different scenarios show possible applications of this method for indoor rescue missions. Iranmanesh et al. proposed heuristic flight path planning (HFPP) that plans a UAV’s flight path based on parcel delivery destination as well as data delivery destinations [33]. Their results showed that HFPP delivers up to 33% more data packets compared with Encounter-Based Routing and Epidemic routing protocols.

DTN networks are useful for providing communication in environments where frequent transmission discontinuities are present. The main objective of this paper is to ensure communication between isolated areas or in areas with technical damage. In these circumstances, we considered a UAV network with a precise travel and rest period. Thus, the interest would not be on having a multi-copy routing protocol, but on establishing the optimal path for the messages. The results obtained by the proposed TD-UAV Dijkstra algorithm, a single-copy algorithm, and multiple-copies algorithms were compared in the case of buffer-limited capacity. A space mapping method using a topological map as a collection of regular polygons is proposed. In addition, another practical utility is to facilitate the transmission of information, materials, or other goods in quarantined regions due to the presence of an outbreak of infections during a pandemic, as is the case of the Coronavirus pandemic. The proposed UAV networks are independent of the Internet and they are of the DTN type. Multiple simulations based on experimental preliminary flight tests are performed to analyze the efficiency of the data transmission in the case of protocols based on the transmission of a single copy or multiple message copies.

## 2. Materials and Methods

### 2.1. Area Mapping Method Using UAVs Flying on Regular Polygons

**Figure 1:**
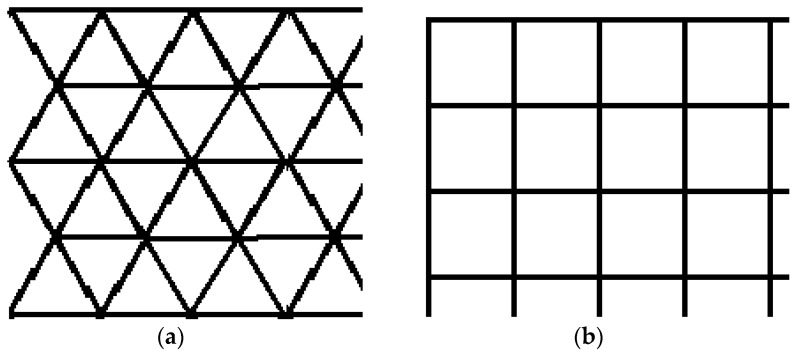
Polygons map: (**a**) Covering a 2D surface with equilateral triangles; (**b**) Covering a 2D surface with squares.

A 2D surface is intended to be covered with points equidistantly distributed using regular polygons. The surface can be covered in this way with points grouped into equilateral triangles or squares (Figure 1).

**Figure 2:**
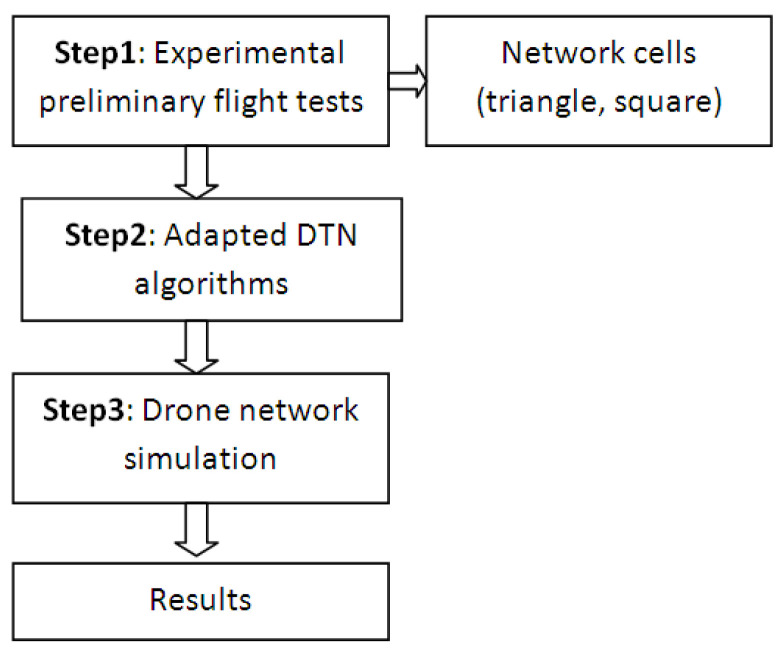
Novel method for preliminary UAV network analysis.

A new method for UAV network analysis and evaluation based on experimental preliminary flight tests is proposed, as shown in the flowchart from Figure 2. The experimental preliminary flight test of the UAV performed under different environmental conditions allows estimation of the UAV performance under real conditions. The results are used as input parameters of simulation for different DTN algorithms.

An environment map is essential for both UAV flight control and in simultaneous localization and mission tasks. There are three map types, as was mentioned in [41], metric, topological, and hybrid maps. A metric map is represented as a grid, geometric, or feature map [37]. Topological maps are represented by graphs comprised of nodes and edges, where nodes represent places, and edges represent the path between nodes [37,41]. A hybrid map consists of small metric map places in nodes, connected by edges that are the paths between metric maps [37]. In this work, a topological type map was proposed.

**Figure 3:**
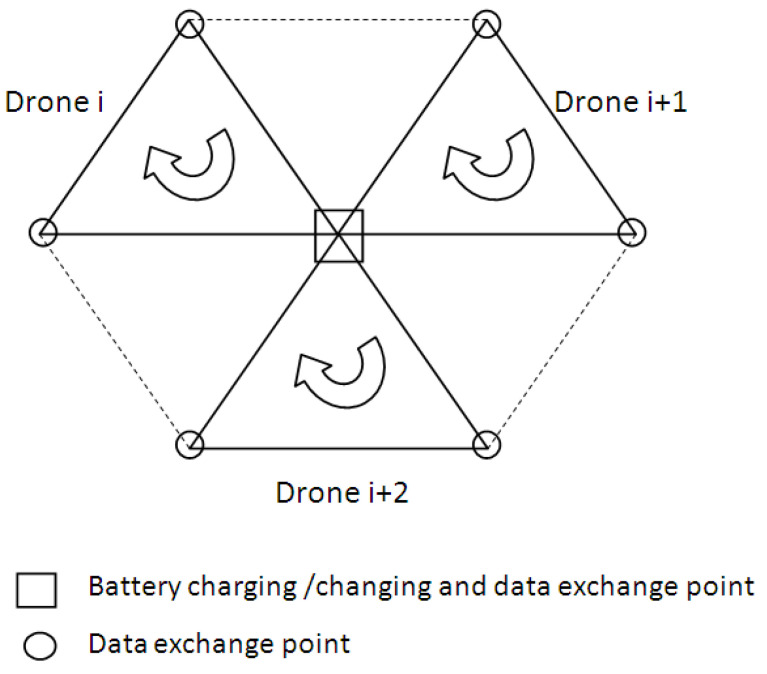
Network cells: hexagonal cell with three UAVs.

To cover a surface, a hexagonal cell comprising three operational equilateral triangles is considered (Figure 3). Each operational cell is covered by a UAV. All the UAVs that operate in the proposed network cells have a specific mission profile. In the middle of each cell, a battery charging/changing station is placed. Thus, in a hexagonal cell, three UAVs share the same battery charging/swapping dock.

**Figure 4:**
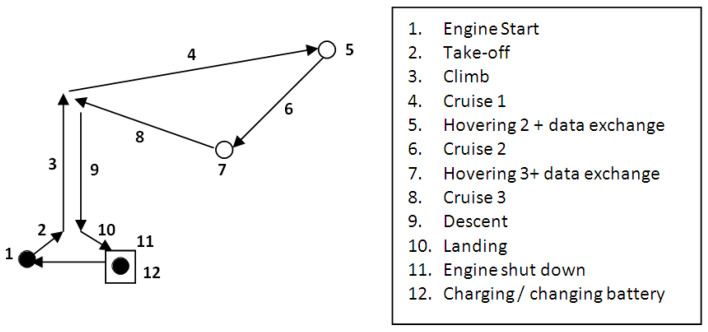
Triangular-shaped flight mission profile of the UAV.

Each flight mission profile consists of several phases or steps. A general flight mission profile of the UAV consists of the following main steps: engine start, take-off, climb to the cruise altitude, cruise, hovering and data exchange, descent, landing, and engine shut-down. The next step consists of charging or swapping the UAV battery followed by a new flight mission. The triangular-shaped flight mission profile of the UAV contains three cruise segments (Figure 4). Each cruise segment is followed by hovering and data exchange steps.

### 2.2. Algorithms for DTNs with UAVs

A DTN network is modeled with a graph. Graph nodes are of two types: fixed and mobile. There are no connections between the fixed nodes, so there is no possibility of data transmission, considering that the distance between the nearest two nodes is 4000 m. Network connections are provided by mobile nodes (UAVs), but their condition is not always the same; they have periods when they are active and periods when they are inactive. This means that there is not always an end-to-end path available between any two nodes in the graph.

**Figure 5:**
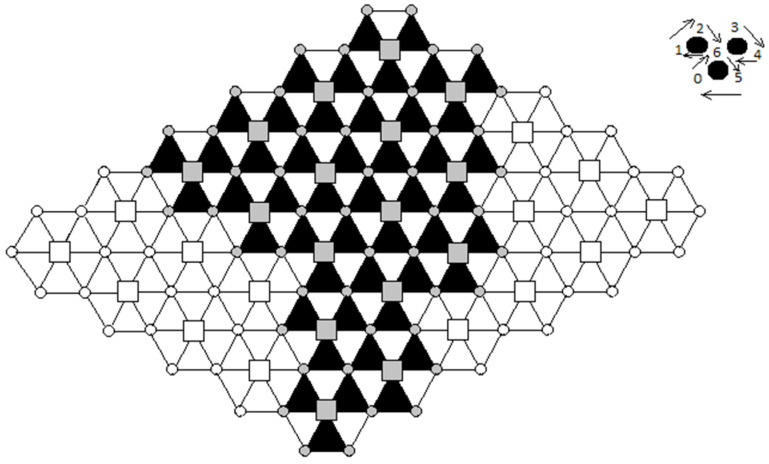
Triangular network considered for experiments.

Five well-known routing algorithms for DTN networks (Epidemic, Spray and Wait, PRoPHET, MaxProp, and MaxDelivery [42]) and the new TD-UAV Dijkstra approach were tested on the triangle-shaped from Figure 5. Tests by choosing random sources and random destinations, which can be located on any node (marked as gray circle or grey rectangle), were performed. It was considered that the UAVs work every day from 7:00 AM to 6:00 PM. The messages can leave a source node between 7:00 AM. and 5:00 PM. A total of 1000 messages randomly sent in this interval of time were considered.

Epidemic is the basic form of a flood-based routing protocol: when two nodes meet, they identify the packages that the other node has and it does not have so that at the end of the process, the two nodes have the same content in the buffer. This process is repeated each time two nodes come into contact. When a node has a copy of a message, it waits to meet the destination. In this case, the resource consumption is high, but in a high mobility network, the delay of the message transmission is short. In the current network configuration, the algorithm produces poor results due to the low number of contacts between nodes.

Spray and Wait is an algorithm with two phases: one for sending messages (spray) and the other waiting for the contact with the destination node (wait). This algorithm circulates in a couple of variants: a standard one and a binary one, depending on the number of spread copies of the message. It acts similar to Epidemic, with an important difference: the number of spread copies is constant. The spray phase of the standard approach consists of spraying L copies of the message by the source node itself. The spray phase of the binary approach consists of spraying half of the number of copies to a meeting node. In this case, not only the source sprays messages, but also every node that has more than one copy does the same. The nodes that have only one copy enter the waiting phase. This algorithm has the disadvantage that nodes must keep track of movements of other nodes, but the advantage is that the level of flooding is limited.

PRoPHET is similar to Epidemic, too, except that it uses information from the buffer of the other node to update its predictability vector. Each node calculates the predictability of the message delivery and sends the message further only if the contact node has higher predictability than its own. The problem of this approach is the relationship between the overhead ratio and the number of nodes—as the number of nodes increases, the overhead ratio increases [16]. This protocol is known for its complexity of the forwarding strategy. Thus, it consumes a lot of resources to process and store historical values. It is feasible for networks with high computation and infrastructure capabilities.

MaxProp is an algorithm based on prioritizing packet transmission and discarding. The packets in the queue are divided into two categories: those below the “n” hop threshold (up to that point) and those above this threshold. Newer packages that have not traveled too far are considered a priority, and the guarantee that they will reach their destination is considered to be high. In this algorithm, there is a need for high computation and infrastructure capabilities. This protocol has low performance when nodes have small buffer sizes because of the adaptive threshold calculation, but it gives better performance with a larger buffer size. It has a so-called slow start problem, because, in the case of a large network, it may take a very long time before each node receives the delivery predictability of other nodes because of the disconnecting nature of the networks, as shown in [21].

MaxDelivery [42] is an algorithm based on prioritizing message delivery, using an appropriate buffer management strategy, consisting of forwarding, dropping, and buffer cleaning mechanisms.

**Figure 6:**
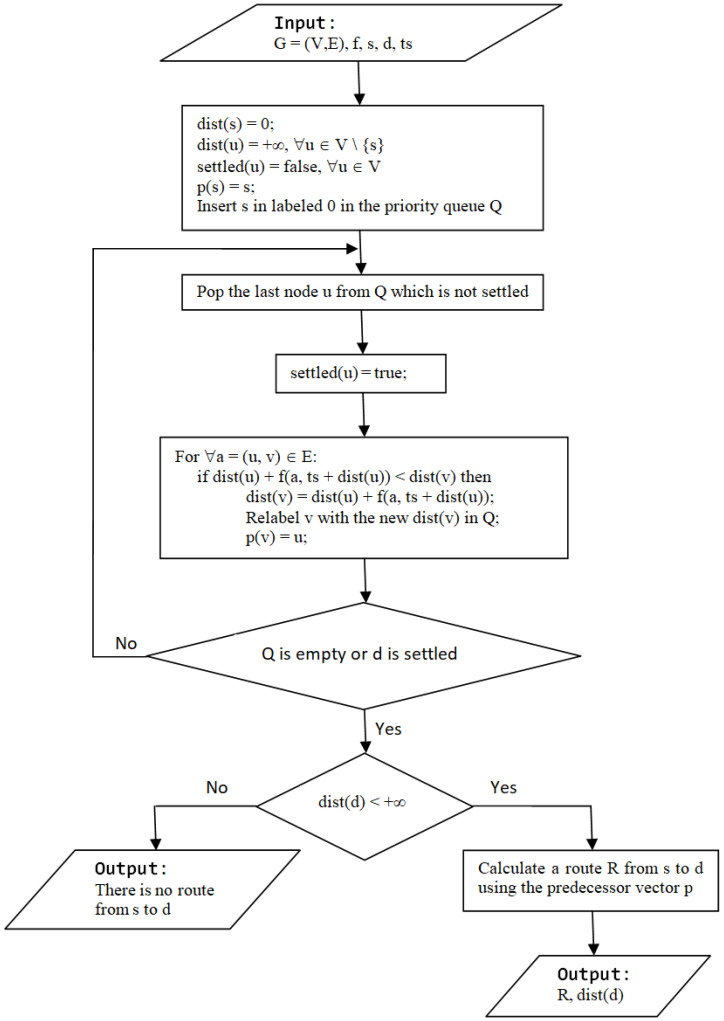
Flowchart of the TD-UAV Dijkstra algorithm.

The Dijkstra algorithm is a method used to find the shortest path connecting two nodes in a network [43]. Our routing problem can be modeled using a time-dependent oriented network [44]. For the triangle drone network, an algorithm named TD-UAV Dijkstra (Figure 6) is proposed to find a route and its distance dist(d) in seconds from the source s to the destination d starting at the moment “ts”. In this network, the nodes (from the set denoted V) are the vertices of the triangles, and the arcs (from the set denoted E) are the connections between these points assured by drones. The length of each arc a = (u, v) is in seconds, is different in time, and depends on the arrival moments of the drone at node v. In the flowchart in Figure 6, f(a, t) denotes the moment of arrival at node v if node u is left at moment t on the arc a = (u, v). The arrival moments of drones for all arcs are pre-calculated (once before starting to use the algorithm) since an exact schedule for drones is known based on each drone’s starting second in a day, the travel on each arc, data transfer at nodes, and wireless charge/battery change time for each drone.

In Table 2, the characteristics of each of the above six algorithms used for simulation are summarized.

**Figure 7:**
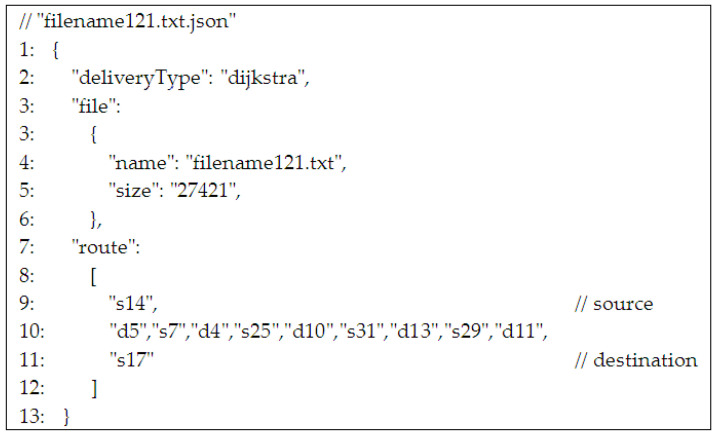
json file that keeps route information for the TD-UAV Dijkstra algorithm.

Delivery protocols of each file that has to be delivered were proposed. Thus, a “json” file is attached that keeps all the information needed to transfer the data file from the source to the destination: delivery type (Dijkstra, Spray and Wait, etc.), file information (name, size), and route information (node ids: stations and UAVs). A “json” file for a Dijkstra-type delivery of a data file named “filename121.txt” is shown in Figure 7. As a convention, in the above example, the stations are encoded starting with “s” followed by the numerical id of the station. Similarly, the UAVs are encoded with “d” followed by the numerical id of the UAV. Of course, each route starts and ends with a station id, each station id (except for destination) is followed by an UAV id, and after each UAV id, a station id is next.

**Figure 8:**
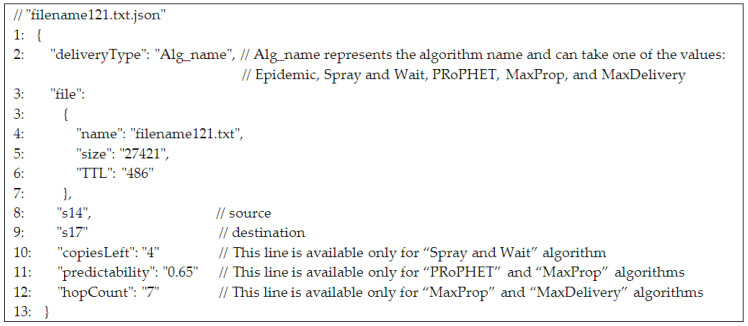
json file that keeps information for the algorithms implemented in the ONE environment.

The json file that keeps information for the algorithms implemented in the ONE environment is presented in Figure 8.

The transfer between the UAV and station is initiated when the UAV is approaching it. All the files that have that station id in the attached json file are transferred, from the UAV to the exchange point. In the case of the Dijkstra algorithm, if the station’s code is the last one in the route enumeration, it means that the file reached the destination. After the UAV transfer to the station is completed, the station transfers to the drone all the files that have the drone’s id in the attached json.

### 2.3. Performance Evaluation by Simulation of the UAV Network

#### 2.3.1. UAV Characteristics and Experimental Flight Tests

A rotary-wing micro-UAV of a quadcopter [45] type was considered in this study. Thus, a DJI Mavic 2 Pro (DJI, Shenzhen, China) UAV was used. The main UAV’s characteristics are presented in Table 3 [46]. A flight altitude of 30 m was chosen based on the literature survey of the micro-UAV missions [47].

The UAVs can be operated remotely or in a pre-programmed way. The proposed UAV network is operated in a pre-programmed way and communicates with data exchange points (with each other) via wireless links. The DJI Mavic 2 Pro quadcopter uses eight high-resolution and two infrared sensors that allow omnidirectional obstacle sensing that determines the relative speed and distance between the UAV and the object and assures good stability in forward and hovering flight. Omnidirectional obstacle sensing includes left, right, up, down, forward, and backward obstacle sensing.

The average charging time of a UAV battery is around 90 min. During this time, the UAV is locked in a UAV charging station. There are some techniques used to allow the battery-based UAVs to increase endurance [48], such as swapping the battery, laser-beam in-flight recharging, solar cells, wireless recharging, and tethered UAVs. An automated way of wire charging of the battery can be performed using a charging platform installed on the ground and one UAV retrofit-kit mounted on the UAV [49,50]. The electricity needed at each station can be provided by a solar panel that charges a battery located at the station. The landing gear of the UAV can be electrically connected by touch with the charging platform after the UAV landing, and charging starts automatically. The main disadvantage of this system consists of locking the UAV on the ground during the battery charging.

A battery swapping and recharging system in an automatic way was proposed to be used for the UAVs, similar to the one from [51]. The battery is automatically recharged after it is swapped at the station. Swapping time depends on the efficiency of the swapping mechanism having values of 15 s [51] to 60 s [52]. A maximum swapping time before the UAV is ready to take off of 60 s is considered.

**Figure 9:**
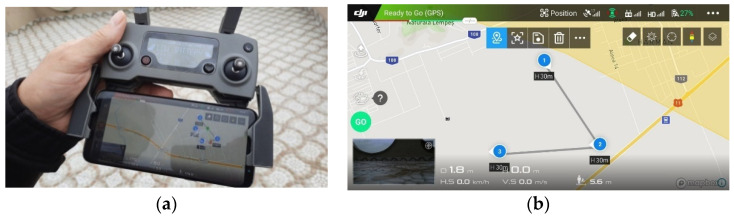
Flight mission segments for preliminary flight tests: (**a**) Remote controller; (**b**) Triangular-shaped flight mission profile.

Usually, in practice, a minimum of 3–5 tests in the design of experiments are considered statistically relevant. Five preliminary flight tests were performed for the triangular-shaped flight mission in the Brasov area in Romania. The results of the flight tests were statistically analyzed, and the average values were used in the simulation of the UAV networks. Experimental flight times for each segment of the UAV flight mission were measured and used in the simulation of the UAV network. A Samsung S8 smartphone device with DJI Go 4 app software (DJI, Shenzhen, China) installed on it, connected to a DJI remote controller (Figure 9), was used to remotely control the UAV.

The preliminary tests were performed at 2 °C temperature, 69% humidity, and 3.5 km/h wind speed. The programming of the flight mission segments for preliminary flight tests was performed within the DJI Go 4 app software.

**Figure 10:**
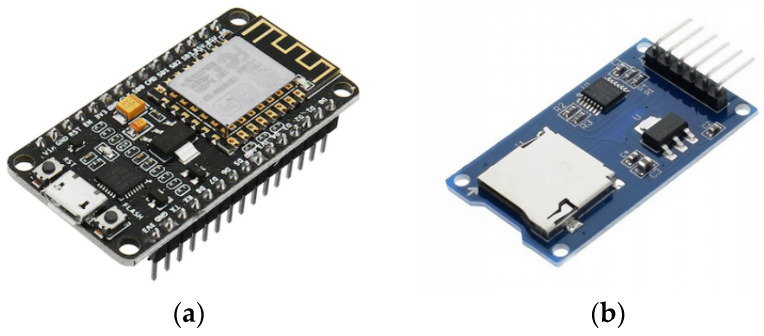
(**a**) NodeMCU Lua Wi-Fi board; (**b**) Micro SD card module.

The transfer between UAVs and stations is assured by a WiFi Arduino development board NodeMCU Lua WiFi, V3, ESP-12E, CP2102 (Espressif Systems, Shanghai, China) [53], Figure 10. ESP-12E offers a complete and self-contained Wi-Fi networking solution. The data (files) are stored on a micro SD card. Both components, the WiFi board, and micro SD card module, are very lightweight, weighing 8 and 5 g, respectively.

The Wi-Fi boards were programmed in Arduino code. The Arduino software allows the writing of programs and uploading them to the Wi-Fi board. The range of the Wi-Fi boards was tested. The connection between them and file transfer was done at a distance of up to 85 m with no obstacles in between. In our model, when transferring files, the UAV was hovering over the station at a height of 30 m which is much less than the maximum distance obtained in range tests. The transfer speed, including writing and reading on/from an SD card, was also tested at a distance of 30 m. An average of 5.81 Mbps was obtained, which makes possible the transfer of a file of 10 MB in 13.77 s.

**Figure 11:**
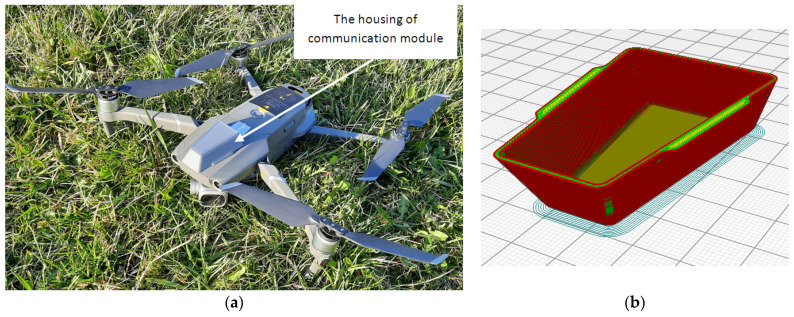
(**a**) DJI Mavic 2 Pro quadcopter with a communication drone-station module; (**b**) 3D model of the communication module case within the 3D printer software simulation.

Housing for the communication module (Figure 11b) was designed using SolidWorks version 2016 software (Dassault Systèmes, MA, USA) to mount and protect the Wi-Fi boards on the UAV. The additive manufacturing machine used for case manufacturing was a BCN3D Sigma R19 (BCN3D Technologies, Barcelona, Spain) using fused filament fabrication (FFF) technology. The material used for case manufacturing was a PLA polylactic acid (BCN3D Technologies, Barcelona, Spain) filament 2.85 mm thick. The Wi-Fi board, micro SD module, SD card, and connection wires together weighed 21.4 g. This load was added to the weight of the UAV, increasing the total weight of the UAV by 2.36%.

#### 2.3.2. Simulation of the UAV Network

When a pandemic (for instance, due to the new COVID-19) occurs, a zone is very likely to be rapidly allocated away from the cities in an isolated area, where ill people are moved. This area, being away from civilization, it is very likely not to have access to any communication systems. In this area, buildings are located with a safe distance between them. In this scenario, each building can be a house where patients are isolated, a warehouse with food or drugs, a laboratory where medical tests are performed for patients, a location where doctors are working (isolated from patients), etc. Data packages (medical images, tests, results, prescriptions from doctors, etc.) must be sent between these buildings. The communication between these buildings can be assured by UAVs organized in a triangular-shaped network. The drone network is kept active until the quarantined area is no longer needed. Of course, when/if direct communication becomes available, the drones could be stopped. The network could be adapted for other purposes such as parcel delivery.

To validate the proposed method, simulations were performed using the UAV network maps as collections of hexagons. Most of the simulations were performed using the Java-based simulator ONE [54,55]. The routing protocols used for the simulation within the ONE simulator are Epidemic, Spray and Wait, PRoPHET, MaxProp, and MaxDelivery.

Based on the characteristics in Table 2, the performance of each of the six considered algorithms was influenced more or less by the parameters in Table 4. For instance, it is clear that the number of fixed transfer points had a greater effect on the algorithms with a limited number of hops, or the average cruise speed and data transmission speed had a greater effect on the algorithms with a short TTL (time to leave for files).

**Figure 12:**
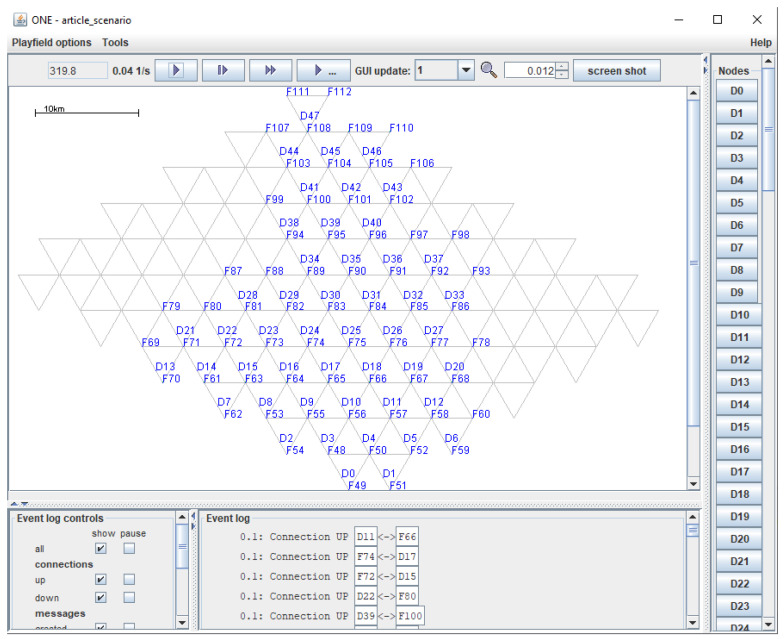
One—triangular network.

All the simulations were performed on an ASUS ROG GL752VW-T4015D laptop with an Intel^®^ Core™ i7-6700HQ 2.60GHz processor and 8 GB of RAM. The main steps within the ONE simulator were as follows:

- Defining the map (Figure 12) in wkt file format, in which the coordinates of all the points that establish the route of each UAV on the map have been defined.
- Implementing the algorithms that define the mobility of UAVs:
  ▪ establishing the initial positions of UAVs and the recharge/swapping points;
  ▪ associating each UAV with a recharger/swapping point;
  ▪ establishing stationary points for data transfer;
  ▪ defining the route of each UAV;
- Establishing the simulation parameters as shown in Table 4. The time parameters (the travel autonomy time, the hovering time for the transfer points, and the parking time) in the charging points or swapping points were established based on the experimental flight tests of the DJI Mavic 2 Pro UAV.

**Figure 13:**
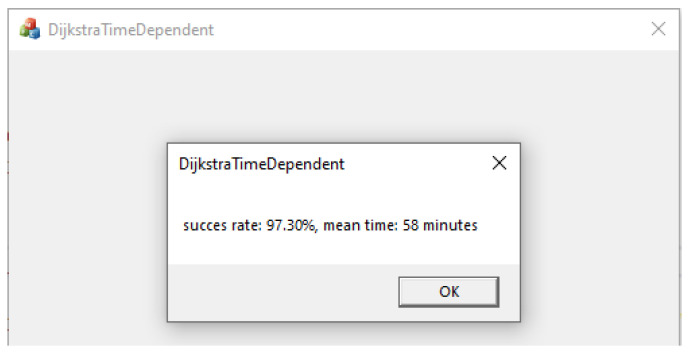
Visual C++ graphical interface: results for triangular networks with battery swapping.

The proposed Dijkstra time-dependent variant was implemented in Visual C++ 2017 programming language. The application has about 1100 lines of C++ source code. The C++ application was executed for each of the two considered situations: triangles with battery charging, and triangles with battery changing. For each case, the same 1000 route simulations from ONE were executed. The graphical interface presenting the results (calculated delivery rate and latency) is shown in Figure 13.

The delivery rate and latency metrics are used to measure the performance of all six routing protocols analyzed in this paper. The delivery rate is determined as a ratio between the number of successfully delivered messages and the number of created ones. The latency is the average time needed for a message to reach the destination starting from the source (departure node).

## 3. Results

### 3.1. Results of Experimental Flight Tests

The theoretical flight times, which can be calculated based on the UAV specifications such as ascent and descent speeds and 50 km/h cruise speed, are not real for simulations. Acceleration and deceleration of the UAV and wind speed are the main factors that influence the experimental flight times. The mean flight time for each flight segment was obtained from experiments (Table 5). In addition, the average cruise speed of the UAV obtained from experiments was 13.16 m/s. The total flight distance of each UAV was 12,000 m.

The percentage of the remaining UAV battery obtained at the end of the flight mission was 23%. The charging time for the UAV battery was 73 min. A safety multiplier of 1.1 was applied to obtain the charging time, and the resulting time was used within the simulations. Thus, instead of 73 min, 80 min were considered in the simulations.

All the results from the experimental flight tests were used within the simulation of the proposed UAV networks.

### 3.2. Simulation Results

This section presents the simulation results of the proposed solution. As discussed earlier, the network area is visualized as a grid with discrete points.

**Figure 14:**
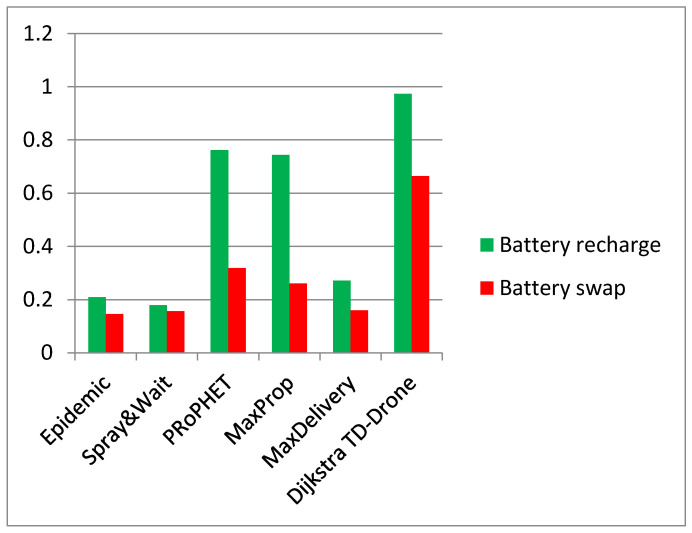
Delivery rate in the UAV network (battery charging vs. battery swapping).

**Figure 15:**
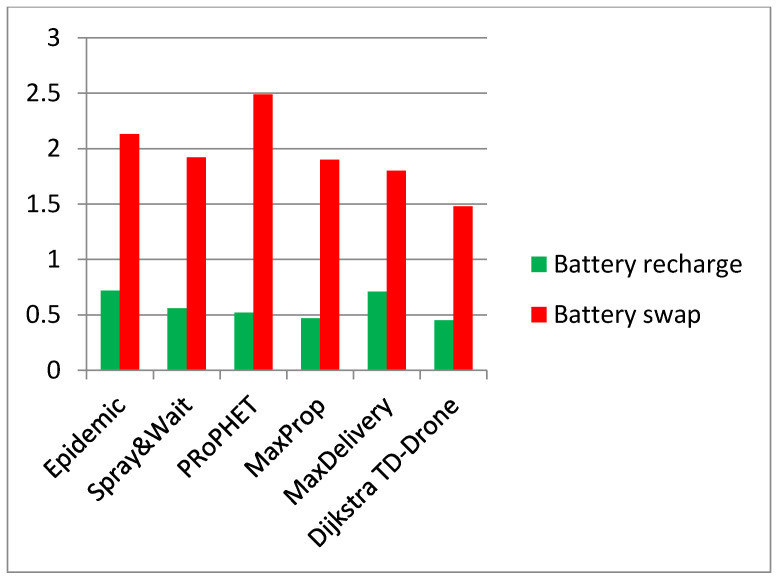
Latency in the UAV network (battery charging vs. battery swapping).

Two performance factors of the DTN UAV network were estimated: delivery rate and average latency. Detailed results of the comparison between battery charge and battery swapping are shown in Table 6. The values of the delivery rate in the case of drone battery swapping are in the range of 0.166 and 0.646 for the routing protocols Epidemic, Spray and Wait, PRoPHET, MaxProp, and MaxDelivery. The best delivery rate is an impressive 0.973 for the TD-UAV Dijkstra protocol (Figure 14). The worst results of latency were obtained for Epidemic, and the TD-UAV Dijkstra average latency was the best, as expected (Figure 15).

The delivery rate in the case of drone battery charge is between 0.135 and 0.143 for the routing protocols Epidemic, Spray and Wait, PRoPHET, MaxProp, and MaxDelivery. The maximum delivery rate is 0.664 and was obtained for the TD-UAV Dijkstra protocol. The worst results for latency were obtained for PRoPHET, and the TD-UAV Dijkstra latency was the best.

The comparison between average latency (Figure 15) was performed on the routes where the delivery was successful for all the algorithms (62 routes for the case of battery swapping and 76 in the case of battery recharge).

## 4. Discussion

The best delivery rate in all cases was obtained using the TD-UAV Dijkstra algorithm since its buffer load was minimum (the data were loaded only in the stations and on UAVs belonging to the calculated route). The latency in the case of the Dijkstra algorithm was high since it delivered most of the packages. Generally, the other algorithms could only deliver over shorter distances due to buffer restrictions (Table 4). It is known that the Dijkstra algorithm assures the shortest time delivery and, therefore, the best latency.

The algorithms Epidemic, Spray and Wait, PRoPHET, and MaxProp are classical for DTN, but in our case, as shown, the Dijkstra algorithm time-dependent adaptation was successfully used since the flight timetables were known. The advantages of the TD-UAV Dijkstra algorithm are an exact and optimum route that is a priori calculated assuring the fastest time of delivery from departure to destination if the route exists; a message is not uselessly sent in the network if no route exists from departure to destination, multiple copies of the messages are not uselessly spread through UAVs and station buffers, resulting in useless overloading of the buffers, and of course the rate of delivery success is maximum. The drawback of the TD-UAV Dijkstra algorithm is that the route was calculated using the information about the operating drones and stations at the moment of route calculation and if a drone or a station from the route is down on this route, the message does not reach the destination. All the routes passing through the station or drone that is down are compromised until the fault is detected. Moreover, this problem reappears when the drone/station is fixed until the moment this information is updated. However, the chance of this problem is low and if it appears, it is fixed in time after the drone/station is repaired or the current status of the network is updated. Using, for instance, the Spray and Wait or Epidemic algorithms presented in this paper, any message has the chance to reach the destination even if drones or stations are down since the copy of the message is spread in the network.

## 5. Conclusions

This paper presented a novel method for communication in quarantined areas, e.g., due to the new COVID-19 pandemic, between isolated areas and in areas with technical damage, using UAV networks with a flight mission plan on triangular cells and with a well-established mobility schedule. Two main scenarios of drone battery management using charging and swapping battery stations were investigated. The following conclusions are drawn:

- A novel method for mapping an area using regular polygons was proposed. The proposed network of cells to cover a geographical area is hexagonal, each having three UAVs.
- A new methodology based on experimental preliminary flight tests for a network cell was proposed to simulate a UAV cell network.
- A new TD-UAV Dijkstra algorithm and well-known DTN algorithms were analyzed to simulate UAV networks with a well-established mobility schedule.
- A delivery rate of 0.146 to 0.644 in the UAV network with a respective battery charge of 0.179 to 0.973 with battery swapping was found. The best results were obtained for the TD-UAV Dijkstra algorithm, which delivered most of the data packages in the shortest delivery time. The average latency was 1.48 h for the UAV network with battery recharge and 0.45 h for the UAV network with battery swapping.
- The Epidemic, Spray and Wait, and MaxDelivery algorithms produced poorer results due to the small number of contacts between nodes and a low number of message exchanges.
- The fastest communication was obtained for a UAV triangular network with a battery charge. It was found that the battery swapping scenario led to an increase of ~46% for the delivery rate against the battery charge scenario.

A future direction of research for this type of UAV network with a well-established mobility schedule would be to use them for parcel delivery in emergencies in remote quarantined zones. In addition, a square cell network could be considered instead of the triangular one since both networks can be constructed starting from the idea of equidistantly covering a given 2D area with points that are vertices of some regular polygons (see Figure 1a,b). Both methods could be compared to determine which is better under various conditions.

## Figures and Tables

**Table 1:** UAV applications.

Applications	Reference	Objective	Network
Meteorology	[27]	Obtain information about near-surface thermodynamic fields	No
	[28]	Measure the temperature and relative humidity	No
	[29]	Measure the wind speed and wind direction	No
Rescue mission	[37]	UAVs perform the building and exploration of a honeycomb map	Yes
Communications	[30]	Investigate the UAV-based air-to-ground-radio vortex wireless networks	Yes
	[31]	Routing in a vehicular ad hoc network (VANET)	Yes
Transportation	[33]	Exchange data packets during a contact such that the data Delivery delay decreases and the delivery ratio increases	Yes
	[34]	Study the potential of delivery and passenger drones	No
Agriculture	[35]	Coordinate drone movements in order to perform adequate count measures against parasite attacks	Yes
Cartography/photogrammetry	[36]	Aerial image acquisition and processing	No

**Table 2:** Algorithm characteristics.

Algorithm	Characteristics
Epidemic	TTL
Spray and wait	TTL, maximum allowed number of copies
PRoPHET	TTL, predictability
MaxProp	TTL, predictability, hop count
MaxDelivery	TTL, hop count
Dijkstra	drone timetable

**Table 3:** UAV parameters [46].

Parameter	Value
Dimensions	214 × 91 × 84 mm (length × width × height)
Max. Ascent/Descent Speed	4 m/s; 3 m/s
Max. flight time (no wind)	31 min (at a consistent 25 km/h)
Max. flight distance (no wind)	18 km (at a consistent 50 km/h)
UAV battery	3850 mAh, 1800 mA, 3.83 V
Weight with battery	905 g
Approx. price	1600 USD
Operating Temperature Range	0–40 °C

**Table 4:** Simulation parameters.

Parameter	Triangular-Shaped Flight Mission
Number of UAVs for cruising	48
Number of fixed transfer points	65
Number of charging/changing battery points	24
Average cruise speed of a UAV	47.37 km/h (13.16 m/s)
Flight height of UAVs	30 m
Operating time of the UAV in one day	11 h
Data transmission speed	2 Mbps
UAV’s buffer space	2 Gb
Message size	500 kb–1 Mb
Message time to live	10 h
Source and destination of messages	any UAV
No. of route simulations	1000

**Table 5:** Flight test results.

Mission Phase	Experimental Mean Flight Time [s]	Standard Deviation
Take off + Climb (30 m)	8.24	0.193
Cruise_segment (4000 m)	304	0.352
Transfer data	120	-
Descent + Landing (30 m)	12.12	0.085
Total flight on triangular cell	1172	1.127

**Table 6:** Efficiency factors in the drone network.

Algorithm	Delivery Rate		Latency (h)	
	Battery Swapping	Battery Charging	Battery Swapping	Battery Charging
Epidemic	0.209	0.146	0.72	2.13
Spray and Wait	0.179	0.156	0.56	1.92
PRoPHET	0.762	0.319	0.52	2.49
MaxProp	0.743	0.261	0.47	1.90
MaxDelivery	0.271	0.160	0.71	1.80
TD-UAV Dijkstra	0.973	0.664	0.45	1.48

## Data Availability

Not applicable.
